# The dynamics of novel corona virus disease via stochastic epidemiological model with vaccination

**DOI:** 10.1038/s41598-023-30647-3

**Published:** 2023-03-07

**Authors:** Rahman Ullah, Qasem Al Mdallal, Tahir Khan, Roman Ullah, Basem Al Alwan, Faizullah Faiz, Quanxin Zhu

**Affiliations:** 1 MOE-LCSM, School of Mathematics and Statistics Hunan, Normal University, Changsha, 410081 China; 2 Department of Mathematical Sciences, UAE University, P. O. Box 15551, Al-Ain, UAE; 3 Department of Mathematics, Women University Swabi, Swabi, 23430 Pakistan; 4 Department of General Studies, Higher Colleges of Technology, Dubai Women Campus, Dobai, UAE; 5 Chemical Engineering Department, College of Engineering, King Khalid University, Abha, 61411 Saudi Arabia; 6 Department of Basic Sciences and Humanities, College of Electrical and Mechanical Engineering, National University of Sciences and Technology (NUST), Islamabad, Pakistan

**Keywords:** Applied mathematics, Statistics

## Abstract

During the past two years, the novel coronavirus pandemic has dramatically affected the world by producing 4.8 million deaths. Mathematical modeling is one of the useful mathematical tools which has been used frequently to investigate the dynamics of various infectious diseases. It has been observed that the nature of the novel disease of coronavirus transmission differs everywhere, implying that it is not deterministic while having stochastic nature. In this paper, a stochastic mathematical model has been investigated to study the transmission dynamics of novel coronavirus disease under the effect of fluctuated disease propagation and vaccination because effective vaccination programs and interaction of humans play a significant role in every infectious disease prevention. We develop the epidemic problem by taking into account the extended version of the susceptible-infected-recovered model and with the aid of a stochastic differential equation. We then study the fundamental axioms for existence and uniqueness to show that the problem is mathematically and biologically feasible. The extinction of novel coronavirus and persistency are examined, and sufficient conditions resulted from our investigation. In the end, some graphical representations support the analytical findings and present the effect of vaccination and fluctuated environmental variation.

## Introduction

A severe outbreak started in Wuhan, China, at the end of 2019^1,2^. The causative agent was the novel coronavirus, also known as the severe acute respiratory syndrome coronavirus 2 (SARS-CoV-2). It was identified in January 2020 by isolating a single patient. The SARS-CoV-2 pandemic infected 589 million people, as confirmed, and produced 6.5 million deaths up to August 2022 around the world. WHO has declared that it is an international public health emergency. The SARS-CoV-2 virus is highly infectious due to rapid spreading around the globe, so a global pandemic occurs. The potential spreading of the disease is a complex issue for public health. Typical symptoms of SARS-CoV-2 infection include cough, fever, fatigue, breathing difficulties, etc. Vaccination plays a vital role in disease prevention. Recently after the pandemic of the SARS-CoV-2 virus, there are various vaccines available, and ultimately the immunization programs go massively to reduce the depreciation of the novel coronavirus disease.

Mathematical modelling is one of the useful methods and emerging area in the field of applied sciences^3–6^. In the last few decades, mathematical modeling has been used frequently to study the dynamics of real-world problems^7–10^. The modeling approaches are used to explain the complex behavior of infections and non-infectious diseases^11–14^. Various researchers have developed models to understand and monitor the spread of infectious diseases and predict their future forecasting. A mathematical model applied to investigate the dynamics of hepatitis B in a high-prevalence population^15^. Zou et al., proposed a model for the transmission dynamics and control of hepatitis B virus in China^16^. The model of cholera with some interventions is presented in^17^. A comparison of susceptible-infected-recovered and susceptible-exposed-infected-recovered models has been discussed in^18^. The analytical and approximated solution of the stomach model has been studied in^19^. To solve the HIV infection model with latently infected cell a Morlet neural networks model and heuristic approach has been presented (see for more detail^20,21^). The Nonlinear mathematical model that represents the smoking dynamics (has been discussed) with an advanced heuristic approach, and Gudermannian neural networks^22,23^. The artificial network scheme for the solution of the nonlinear model (describes) the schematic process of influenza disease has been studied in^24^. Moreover, based on epidemiological models, effective control mechanisms are forecasted to suggest useful guidelines for health officials and take serious steps to control various infectious diseases. The control of novel coronavirus is a challenging task and attracts the attention of various researchers.

Modeling the dynamics of novel coronavirus has a rich literature, and numerous studies are investigated. A modeling study reported forecasting the potential spread of the 2019-novel pandemic originating in Wuhan city of China^25^. A deep learning algorithm has been used to study the host and infectivity prediction of a novel coronavirus in Wuhan^26^. The transmission of novel coronavirus infection from an asymptomatic contact studied by Rothe et al.^27^. The analysis and prediction of covid-19 epidemic trend have been discussed with the combination of Markov and LSTM method in^28^. Tao et al.^29^ discussed the summary of the covid-19 epidemic with estimating the effects of emergency responses in China. Moreover, the role of asymptomatic, quantifying the effect of remdesivir in rhesus macaques infection, and characterization of the recent pandemic with the impact of uncertainties are respectively discussed in^30–32^. Aguiar et al. also present a model to study the critical fluctuations of covid-19 via epidemiological model^33^. Similarly, many other models were reported for investigating SARS-CoV-2 virus dynamics (see for instance^34–36^).

The literature shows that the reported epidemiological models are described with the aid of a deterministic differential equation, but SARS-CoV-2 virus transmission is not deterministic while influenced by different factors and therefore has stochastic nature. Nevertheless, the reported studies produce valuable outputs; however, modeling with the aid of stochastic differential equations is more appropriate and can produce accurate results compared to the deterministic differential equation models^37–41^. Particularly, a stochastic model with non-linear incidence has been investigated to study the extinction analysis and stationary distribution^42^. Zhou et al., discussed an epidemic model with stochastic perturbations in^43^. The threshold behavior of a model is presented via a stochastic epidemiological model^44^. The main contribution of this paper is to formulate an alternative stochastic epidemiological model for the SARS-CoV-2 virus with the aid of a stochastic differential equation and to incorporate the fluctuated disease prorogation as well as including the effect of vaccination according to the characteristic of the disease.

**Figure 1:**
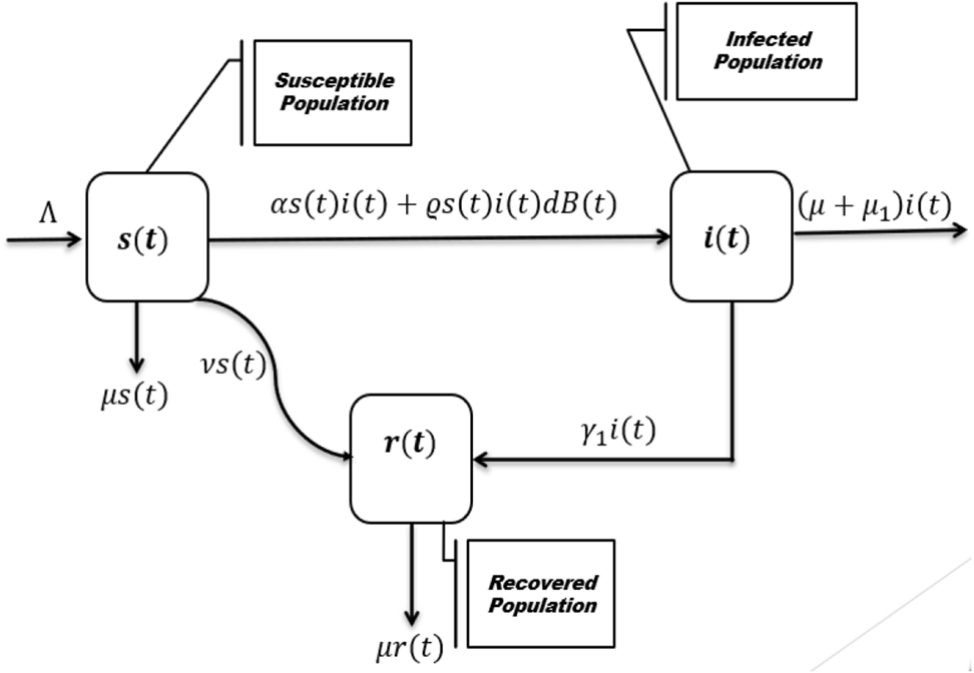
The graph demonstrate the evolution and schematic process of the novel corona virus propagation.

We propose a stochastic mathematical model to study the dynamics of the SARS-CoV-2 virus in a population under the effect of vaccination and fluctuated disease propagation. For this purpose, the various compartment population is as *s*(*t*), the susceptible, *i*(*t*), the infected with SARS-CoV-2 virus, and *r*(*t*), the recovered, where the transmission rate of the disease is divided by the white noise to incorporate the environmental variation, see the schematic process of the novel coronavirus evolution in Fig. 1. The model proposes population classes that are linked to the identical information source and given by the Brownian motion filtration. It is also worth mentioning that from the characterization of the transmission of the SARS-CoV-2 virus transmission rate is different everywhere, so the random fluctuation is assumed to incorporate the stochastic effect. We also assume that the successful vaccination of susceptible individuals will be got permanent immunity. We then prove the model’s well-posedness with the aid of stochastic Lyapunov function theory and perform the extinction and persistence analysis to find sufficient conditions for the novel coronavirus elimination. We also perform some numerical simulations to show the feasibility of the obtained results with the help of the Maruyama method.

## Organization

The organization of the paper is as follows: we derive the governing equation for the proposed model in section “Model formulation” and discuss the detailed mathematical as well as biological feasibility of the model in section “Existence and uniqueness analysis”. We discussed the disease extinction and persistence to obtain some sufficient conditions in section “The analysis of extinction and persistence”. Further, the numerical simulations of the model have been performed in section “Simulation analysis”. We end with the conclusion in the section “Conclusion”.

## Model formulation

We present the proposed stochastic model: susceptible-infected and recovered model under the effect of vaccination and varying population by placing the following constraints in terms of some assumptions.

1. All parameters, variables, and constants of the problem are non-negative values.
2. The newborn is susceptible.
3. We symbolize the total population by *x*(*t*) divided into various population groups of susceptible, infected with SARS-CoV-2, and recovered. Mathematically we can write as $x(t)=s(t)+i(t)+r(t)$ as varies against *t*.
4. The pandemic of SARS-CoV-2 rises due to human contact. The transmission everywhere differs; therefore, the various groups of the model are driven by the equal randomness or source as symbolized by *B*(*t*). The change in these populations groups is associated with the same information source demonstrated by the Brownian motion filtration $F=(F_t)_{t\in [,T]}$, where $F_t:= \sigma (B(t))$ is the s-algebra as generated by the Brownian motion *B*(*t*). The results show that a variation in a group has an impact directly on the other group. We fluctuate the disease transmission parameter in such a way that $\alpha \rightarrow \alpha +\varrho {\dot{B}}(t)$, where the standard Brownian motion is symbolized by *B*(*t*) is and so $B(0)=0$, and $\varrho$ is the white noise intensity satisfying $\varrho ^2>0$.
5. The vaccination of susceptible populations will be accorded permanent immunity.

Thus, the governing equations of the models ultimately take the following form.

1 $$\begin{aligned} \left\{ \begin{array}{l} \displaystyle ds(t)=\big \{\Lambda -s(t)(\alpha i(t)+\mu +v)\big \}dt-\varrho i(t)s(t)dB(t),\\ \displaystyle di(t)=i(t)\big \{\alpha s(t)-\gamma _1-\mu _1-\mu \big \}dt+\varrho i(t)s(t)dB(t),\\ \displaystyle dr(t)=\big \{vs(t)+\gamma _1i(t)-\mu r(t)\big \}dt, \end{array} \right. \end{aligned}$$

where the detailed characterization of the model parameters is as we use $\Lambda$ as a parameter for the newborn rate, while the death rate (natural and disease) are symbolized by $\mu$ and $\mu _1$ respectively. The vaccination rate of susceptible is assumed to be *v*, and $\gamma _1$ is the natural recovery or the recovery due to treatment. Moreover, $\varrho$ is the environmental white noise intensity, while *B*(*t*) denotes the scalar standard Brownian motion.

If we put $\varrho =0$, the system (1) reduces to its associated deterministic form, then solving at steady states, we ultimately derive the equilibria (disease-free and endemic) as given by $e_0$ and $e_{*}$ whose components are given

2 $$\begin{aligned} \left\{ \begin{array}{l} e_0=\big (s_0,0,r_0\big ),~e_{*}=\big (s_{*},i_{*},r_{*}\big ),\\ s_0=\Lambda /\big (\mu +v\big ),~r_0=v\Lambda /\big (\mu (\mu +v)\big ),\\ s_{*}=\big (\gamma _1+\mu +\mu _1\big )/\alpha , ~i_{*}=\big (\mu +v\big )\big (\gamma _1+\mu +\mu _1\big )\big [R_0-1\big ]/\alpha ,\\ r_{*}=\big (vs_{*}+\gamma _1i_{*}\big )/\mu , \end{array} \right. \end{aligned}$$

Where $R_0$ is the threshold quantity (*basic reproductive number*) represents the expected number of secondary infections produced by a single infective whenever introduced into a susceptible population. Consequently, calculated with the aid of the next-generation method approach, which becomes

3 $$\begin{aligned} \begin{aligned} R_0=\alpha \Lambda /(\mu +v)(\gamma _1+\mu +\mu _1). \end{aligned} \end{aligned}$$

It could be observed that either the epidemic rises or dies out depending on the value of the *basic reproductive number*. Here we will discuss a brief sensitivity analysis of the *basic reproductive number*. Which provides the relative impact of the model parameters and threshold quantity. Generally, the sensitivity index of a parameter $\psi$ is denoted by $S_{\psi }$ and defined by the following equation

4 $$\begin{aligned} S_{\psi }=\frac{\psi }{R_0}\frac{\partial R_0}{\partial \psi }. \end{aligned}$$

**Figure 2:**
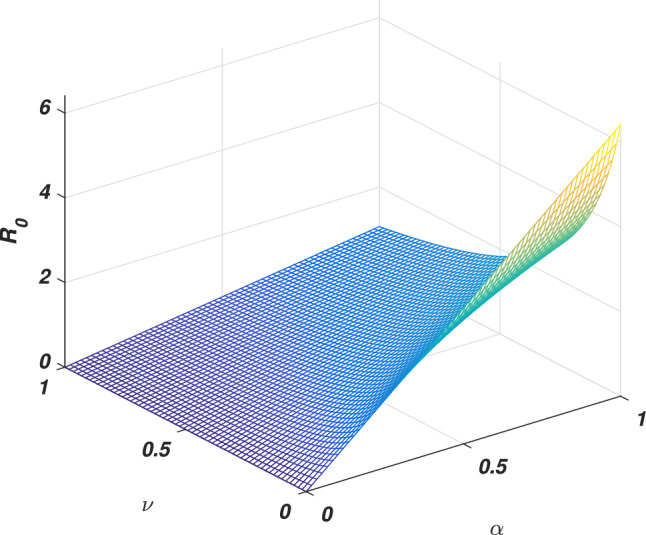
The graph shows the variation of the *basic reproductive number* against the parameters $\alpha$ and *v*.

**Figure 3:**
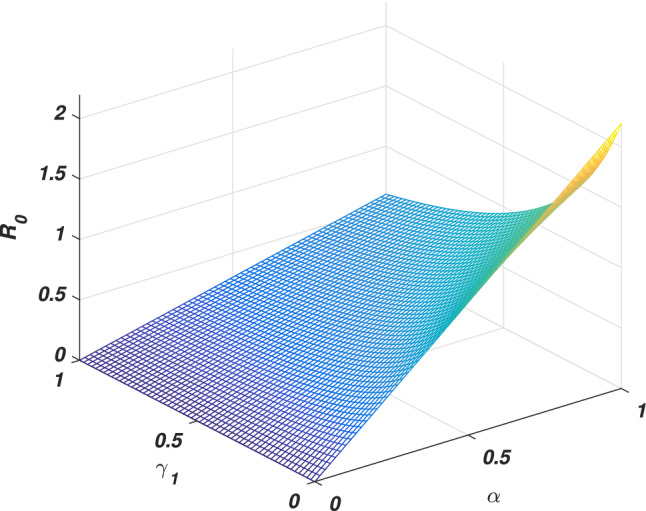
The graph demonstrate the variation of the *basic reproductive number* against parameters $\alpha$ and $\gamma _1$.

**Figure 4:**
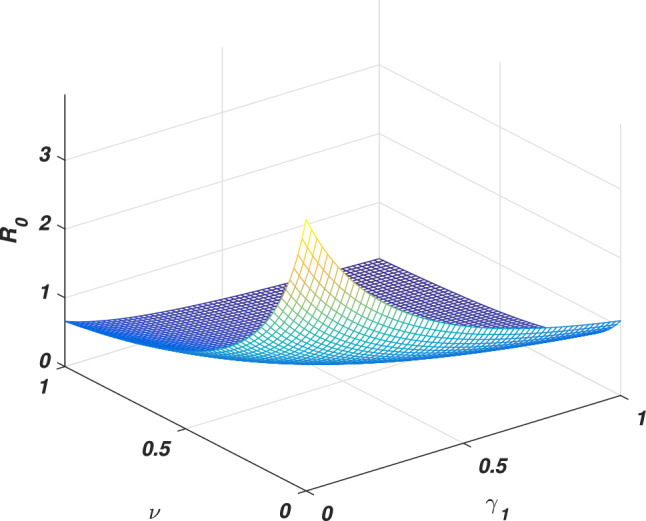
The variation of the *basic reproductive number* for the parameters $\gamma _1$ and *v*.

Let us assume $\Lambda =0.5$, $\alpha =0.6$, $\mu =0.2$, $v=0.4$, $\mu _1=0.18$ and $\gamma _1=0.01$, Eq. (4) gives the sensitivity index of $\alpha$, *v* and $\gamma _1$ are $S_{\alpha }=1$, $S_{v}=-0.67$ and $S_{\gamma _1}=-0.0256$ respectively. And having a direct relationship between $\alpha$ has directly proportional to the threshold quantity while *v* and $\gamma _1$ are inversely proportional. Moreover, $\alpha$ got the highest sensitivity index which implies that if the value of $\alpha$ is increased, or decreased, say by 10% would increase or decrease the value of the threshold quantity by 10%, see Figs. 2 and 3. On the other hand, the second highest sensitive parameter is *v* got the $-0.67$ sensitivity index. That’s increasing the value of *v* say, by 10% would decrease the value of $R_0$ by 6.7% while decreasing may cause increasing as depicted in Fig. 4. Similarly, the sensitivity index of $\gamma _1$ is -0.0256. Which is inversely proportional to the *basic reproductive number*, and so an increase in the value of this parameter decreases the value of the *basic reproductive number*. If the value of $\gamma _1$ is increased or decreased by 10%, would decrease or increases the value of the *basic reproductive number* by 0.256% as shown in Fig. 3. Thus the sensitivity analysis reveals that the two parameters $\alpha$ and *v* are more sensitive, and special attention is required to minimize the contagious infection. Based on this, we suggest that isolation and speedy vaccination are effective control measures to control the transmission of SARS-CoV-2 virus in the community.

## Existence and uniqueness analysis

We show that the model (1) has a unique solution, positive as well as global, and therefore the proposed problem (1) is well-posed. Regarding the existence and uniqueness analysis, we have the following theorem.

### Theorem 4.1

*The solutions to the proposed problem, as stated by Eq.* (1) *is unique for any initial conditions in* $R^{3}_{+}$. *Moreover, the solution will also remain in* $R^{3}_{+}$ *with probability* 1, *namely*, $(s(t),i(t),r(t))\in R^{3}_{+}$ *for* $t\ge 0$ *a.s* (*almost surely*).

### Proof

It could be noted from the proposed problem that the co-efficient of all equations are continuous and locally Lipschitz for $(s_0,i_0,r_0)$ in $R^{3}_{+}$ by following^45^, then there exists a solution on the interval of $t\in [0,\tau _e)$ with the explosion time $\tau _e$. It is now required to investigate whether the solution satisfies the global properties. We will prove $\tau _e=\infty$ a.s. We assume that $k_0$ is non-negative and sufficiently large, then $s_0$, $i_0$, and $r_0$ lie in $\big [\frac{1}{k_0},k_0\big ]$. Moreover, for every *k* i.e., $k\ge k_0$, the stopping time is defined by

5 $$\begin{aligned} \tau _k=\left\{ \min \{s(t),i(t),r(t)\}\le \frac{1}{k}~\text {or}~\max \{s(t),i(t),r(t)\},t\in [0,\tau _e)\right\} . \end{aligned}$$

Let $\phi$ is a null set, then we put $inf\phi =\infty$. It is clear that increase in $\tau _k$ is related to *k* as $k\rightarrow \infty$, then it is increasing and therefore making the substitution of $\tau _\infty =\lim _{k\rightarrow \infty }$ implies that $\tau _\infty \le \tau _e$ a.s. We will prove that $\tau _\infty =\infty$ for investigating $\tau _e=\infty$ and so (*s*(*t*), *i*(*t*), *r*(*t*)) in $R^{3}_{+}$ a.s., for all non-negative *t*. On the other hand, it is needed to show that $\tau _e=\infty$ a.s. However, if this is not true and the statement becomes false, then pair of constants i.e., $T>0$ and $\epsilon \in (0,1)$ exist which satisfy

6 $$\begin{aligned} P\{\tau _\infty \le T\}>\epsilon . \end{aligned}$$

So an integer $k_1\ge k_0$ exists and

$$\begin{aligned} P\{\tau _k\le T\}\ge \epsilon ,~\text {for~all}~k\ge k_1. \end{aligned}$$

It is also be noted from *x*(*t*), that

7 $$\begin{aligned} dx(t)/dt+\mu x(t)\le \Lambda , \end{aligned}$$

which implies

8 $$\begin{aligned} x(t)\le \left\{ \begin{array}{ll} \Lambda /\mu ,&{}\quad \text {if }~x(0)\le \Lambda /\mu ,\\ x(0), &{}\quad \text {if }~x(0)>\Lambda /\mu , \end{array}\right. :=M. \end{aligned}$$

Defining a $C^{2}$-function by *G* i.e., $G:R^3_{+}\rightarrow R_{+}$ as

9 $$\begin{aligned} G=s+i+r-\log s-\log i-\log r-3. \end{aligned}$$

Obviously $G\ge 0$ as $-\log q+q-1\ge 0$ for every $q>0$. Choosing $T>0$ and $k\ge k_0$ are two arbitrary constants, then It${\hat{o}}$ formula for Eq. (9) gives

10 $$\begin{aligned} \begin{aligned} dG&=LGdt+\varrho (i-s)dB(t), \end{aligned} \end{aligned}$$

where

11 $$\begin{aligned} \begin{aligned} LG&\le \Lambda +\alpha i+\mu +v+\frac{1}{2}\varrho ^2(s^2+i^2) +\mu +\mu _1+\gamma _1+\gamma _1i+vs+\mu . \end{aligned} \end{aligned}$$

Using Eq. (8) in Eq. (11) may gives the following assertion

12 $$\begin{aligned} \begin{aligned} LG&\le \Lambda +3\mu +v+\varrho ^2M^2+(\alpha +v+\gamma _1)M +\mu _1+2\mu +\gamma _1:=K. \end{aligned} \end{aligned}$$

Hence

13 $$\begin{aligned} \begin{aligned} E\left[ G(s(T\wedge \tau _k),i(T\wedge \tau _k),r(T\wedge \tau _k))\right]&\le G(s_0,i_0,r_0)+E\left[ \int ^{T\wedge \tau _k}_0Kdt\right] ,\\&\le G(s_0,i_0,r_0)+TK. \end{aligned} \end{aligned}$$

Putting $\Omega _k={\tau _k\le T}$ whenever $k\ge k_1$ then using Eq. (6) imply that $P(\Omega _k)\ge \epsilon$. It is also noted that for all $\omega$ in $\Omega _k$, there exists $s(\omega ,\tau _k)$, $i(\omega ,\tau _k)$, $r(\omega ,\tau _k)$ at least once that is equal to $\frac{1}{k}$ or *k*, and so $G(s(\tau _k),i(\tau _k),r(\tau _k))$ is not less then $-\log k+k-1$ or $-1+\frac{1}{k}+\log k$. Consequently we can write

14 $$\begin{aligned} G(s(\tau _k),i(\tau _k),r(\tau _k))\ge E\big (-\log k+k-1\big )\wedge \left( \log k+\frac{1}{k}-1\right) . \end{aligned}$$

Therefore, Eqs. (6) and (13) implies that

15 $$\begin{aligned} \begin{aligned} G(s_0,i_0,r_0)+TK&\ge E\left[ 1_{\Omega (\omega )}G\big (s(\tau _k),i(\tau _k),r(\tau _k))\big )\right] \\&\ge \epsilon \left[ (-\log k+k-1)\wedge \left( \frac{1}{k}+\log k-1\right) \right] , \end{aligned} \end{aligned}$$

where $1_{\Omega (\omega )}$ is used to represent the *indicator function* of $\Omega$. If $k\rightarrow \infty$ then $\infty >H\big (s_0,i_0,r_0\big )+TM=\infty$ occurs, which shows that $\tau _\infty =\infty$ a.s.

Further, it could be investigated that the solutions of the proposed problem are positive, so the first equation of the system (1) gives the solution

16 $$\begin{aligned} \begin{aligned} s(t)&=e^{-(\mu +v)t-\int _0^t(\alpha i(u)+\frac{1}{2}\varrho ^2i^2(u))du-\varrho \int _0^ti(u)dB(u)}\\&\quad \times \left[ \Lambda \int _0^te^{(\mu +v)u+\int _0^u(\alpha i(v)+\frac{1}{2}\varrho ^2i(v))dv+\varrho \int _0^ui(v)dB(v)}+s(0)\right] du, \end{aligned} \end{aligned}$$

implies that *s*(*t*) is positive. Similarly the second assertion of the model (1) looks like

17 $$\begin{aligned} \begin{aligned} i(t)&=e^{-(\mu +\mu _1+\gamma _1)t+\int _0^t(\alpha s(u)-\frac{1}{2}\varrho ^2s^2(u))du+\varrho \int _0^ts(u)dB(u)}\\&\quad \times \left[ i(0)+\int _0^t\exp \left( \alpha s(s)i(s)e^{(\mu +\mu _1+\gamma _1)v-\int _0^v(\alpha s(u)+\frac{1}{2}\varrho ^2S^2(u))du-\varrho \int _0^s S(u)dB(u)}\right) \right] dv. \end{aligned} \end{aligned}$$

Clearly Eq. (17) gives that *i*(*t*) has non-negative values. On the same way we can prove that *r*(*t*) is positive. $\square$

### Remark 1

It could be noted from the above result as stated by theorem 4.1 that for any initial conditions in $R_{+}^3$, the unique global solution of system (1) exists a.s. Therefore,

18 $$\begin{aligned} dx(t)\le \big (\Lambda -\mu x(t)\big ). \end{aligned}$$

The above Eq. (18) yields

19 $$\begin{aligned} x(t)\le \Lambda /\mu +exp(-\mu t)\big (x(0)-\Lambda /\mu \big )\big (\Lambda -\mu x(t)\big ). \end{aligned}$$

Clearly $x(t)\le \Lambda /\mu$ a.s, if $x(0)\le \Lambda /\mu$ and the feasible region for our considered model (3) is defined as

20 $$\begin{aligned} \Omega ^{*}=\left\{ (s,i,r):s,r>0,i\ge 0, x\le \Lambda /\mu \right\} . \end{aligned}$$

## The analysis of extinction and persistence

We investigate how the disease could be eradicated, and how the disease persists. More importantly, we find the extinction of the model, and the disease persists. We use some basic definitions and notations that will be used in the upcoming analysis. Let *u*(*t*) be any function; then, the mean value is defined as

$$\begin{aligned} \langle u(t)\rangle =\frac{1}{t}\int _0^tu(r)dr. \end{aligned}$$

We also assume that the threshold quantity (*basic reproductive number*) symbolized by $R_0^S$ for the proposed model (1) is given by

21 $$\begin{aligned} R_0^S=\frac{\Lambda \alpha }{\left( \mu +\mu _1+\gamma _1+\frac{\varrho ^2\Lambda ^2}{2(\mu +v)^2}\right) (\mu +v)}. \end{aligned}$$

Moreover, if

$$\begin{aligned} \lim _{t\rightarrow \infty }\inf \int _0^ti(u)du>0~\text {a.s.}, \end{aligned}$$

For the proposed problem, as stated by Eq. (1) holds, then the novel disease of coronavirus persists in the mean. We describe the analysis regarding the analysis of extinction with the help of the following theorem.

### Theorem 5.1

*If* $R_0^S<1$ *and* $\alpha (\mu +v)>\varrho ^2\Lambda$ *is satisfied, then the following holds for the solutions of the proposed model*

$$\begin{aligned} \lim \log i(t)/t<0,~\hbox { as } t\rightarrow \infty ,~\text {a.s.}, \end{aligned}$$

*which describes that* *i*(*t*) *approaches 0 exponentially and*

22 $$\begin{aligned} \lim s(t)=s^0,~\lim i(t)=0~\text {and}~\lim r(t)=r^0,~\text {as}~t\rightarrow \infty , \end{aligned}$$

*where* $(s^0,0,r^0)$ *is the disease free equilibrium of the deterministic version of the proposed problem.*

### Proof

We integrate both sides of system (1) and obtain

23 $$\begin{aligned} \begin{aligned} \int _0^tds(y)&=\left( \Lambda t-\int _0^t\big (\alpha i(y)+\mu +v\big )s(y)\right) dy-\int _0^t\varrho s(y)i(y)dB(y),\\ \int _0^tdi(y)&=\int _0^t\big (\alpha s(y)-\mu -\mu _1-\gamma _1\big )i(y)dy+\int _0^t\varrho s(y)i(y)dB(y),\\ \int _0^tdr(y)&=\int _0^t(v s(y)+\gamma _1 i(y)-\mu r(y))dy, \end{aligned} \end{aligned}$$

which implies that

24 $$\begin{aligned} \begin{aligned}{}&\frac{s(t)-s(0)}{t}=\Lambda -\alpha \langle i(t)s(t)\rangle -(\mu +v)\langle s(t)\rangle -\frac{\varrho }{t}\int _0^ts(y)i(y)dB(y),\\&\frac{i(t)-i(0)}{t}=\alpha \langle i(t)s(t)\rangle -(\gamma _1+\mu +\mu _1)\langle i(t)\rangle +\frac{\varrho }{t}\int _0^ts(y)i(y)dB(y),\\&\frac{r(t)-r(0)}{t}=\gamma _1\langle i(t)\rangle -\mu \langle r(t)\rangle +v\langle s(t)\rangle . \end{aligned} \end{aligned}$$

The addition of the first two equations of the above system (24) leads to the assertion

25 $$\begin{aligned} \langle s(t)\rangle =\Lambda /(\mu +v)-(\mu +\mu _1+\gamma _1)\langle i(t)\rangle /(\mu +v)+\phi (t), \end{aligned}$$

where

$$\begin{aligned} \phi (t)=-\frac{1}{(\mu +v)t}\big [i(t)+s(t)-i(0)-s(0)\big ]. \end{aligned}$$

Clearly $\phi (t)$ approaches 0 whenever *t* approaches $\infty$. Moreover, again system (1) and It${\hat{o}}$ formula application gives

26 $$\begin{aligned} d\log i(t)=\left[ \alpha s(t)-(\gamma _1+\mu +\mu _1)-\frac{1}{2}\varrho ^2s^2(t)\right] dt+\varrho s(t)dB(t). \end{aligned}$$

Integrating both sides of Eq. (26) and dividing by *t* with some algebraic manipulation, we get

27 $$\begin{aligned} \begin{aligned} \frac{\log i(t)-\log i(0)}{t} \le \alpha \langle s(t)\rangle -(\mu _1+\mu +\gamma _1)-\frac{1}{2}\varrho ^2\langle s(t)\rangle ^2+\frac{\varrho }{t}\int _0^t s(y)dB(y). \end{aligned} \end{aligned}$$

Using the notion $M(t)=\varrho \int _0^ts(y)dB(y)$ and $\varphi (t)=\alpha \phi (t)-\frac{1}{2}\varrho ^2\phi ^2(t)+\frac{\varrho ^2(\mu +\mu _1+\gamma _1)}{\mu +v}\langle I(t)\rangle \phi (t)-\frac{\varrho ^2\phi (t)\Lambda }{\mu +v}$ along with the substitution of Eq. (25) in Eq. (27) give the following expression

$$\begin{aligned} \begin{aligned} \frac{\log i(t)-\log i(0)}{t}&\le \frac{\alpha \Lambda }{\mu +v}-\frac{(\mu _1+\mu +\gamma _1)\alpha }{\mu +v}\langle i(t)\rangle -(\mu +\mu _1+\gamma _1)\\&\quad -\frac{1}{2}\frac{\varrho ^2 \Lambda ^2}{(v+\mu )^2}+\frac{\varrho ^2 \Lambda ^2(\gamma _1+\mu +\mu _1)}{(\mu +v)^3}\langle i(t)\rangle +\varphi (t)+\frac{M(t)}{t}, \end{aligned} \end{aligned}$$

Now making use of $R_0^S$ in the above equation implies

28 $$\begin{aligned} \begin{aligned} \frac{\log i(t)-\log i(0)}{t}&\le -\left( \mu _1+\mu +\frac{1}{2}\frac{\varrho ^2 \Lambda }{\mu +v}+\gamma _1\right) \big (1-R_0^S\big )\\&\quad -\left( \frac{\mu +\gamma _1+\mu _1}{v+\mu }\right) \left( \alpha -\frac{\Lambda \varrho ^2 }{v+\mu }\right) \langle i(t)\rangle +\frac{M(t)}{t}+\varphi (t). \end{aligned} \end{aligned}$$

Putting $\phi (t)=0$, as *t* approaches $\infty$, we arrive at

29 $$\begin{aligned} \lim \sup M(t)/t=\lim \varphi (t)=0~\text {as} t\rightarrow \infty , \text {a.s}. \end{aligned}$$

Thus Eq. (28) can be also re-written as

30 $$\begin{aligned} \begin{aligned} \lim _{t\rightarrow \infty }\sup \frac{\log i(t)}{t}&\le -\left( \mu +\mu _1+\gamma _1+\frac{1}{2}\frac{\varrho ^2 \Lambda }{\mu +v}\right) \big (1-R_0^S\big )\\&\quad -\left( \frac{\mu +\mu _1+\gamma _1}{\mu +v}\right) \left( \alpha -\frac{\varrho ^2 \Lambda }{\mu +v}\right) \langle i(t)\rangle <0~~\text {a.s}. \end{aligned} \end{aligned}$$

If $R_0^S<1$ and $\alpha (\mu +v)>\varrho ^2\Lambda$ hold, then Eq. (30) gives

31 $$\begin{aligned} \lim _{t\rightarrow \infty }i(t)=0~~\text {a.s}. \end{aligned}$$

We show the assertion (22), therefore

32 $$\begin{aligned} d(x(t))=\big (\Lambda -\mu (x(t))-\mu _1i(t)\big )dt. \end{aligned}$$

Solving Eq. (32) which yields

33 $$\begin{aligned} i(t)+r(t)+s(t)=exp(-\mu t)\left( i(0)+r(0)+s(0)+\int _0^t\big (-\mu _1i(y)+\Lambda )\exp (\mu y)dy\right) . \end{aligned}$$

We apply the L’Hospital rule to the above Eq. (33) and substitute Eq. (31), then

34 $$\begin{aligned} \begin{aligned} \lim _{t\rightarrow \infty }(s(t)+r(t))=\Lambda /\mu . \end{aligned} \end{aligned}$$

The solution of the limiting system of the 1st equation of model (1) gives

$$\begin{aligned} \lim _{t\rightarrow \infty }s(t)=\Lambda /(\mu +v)~~\text {a.s}. \end{aligned}$$

Similarly it could be obtained that

$$\begin{aligned} \lim _{t\rightarrow \infty }r(t)=v\Lambda /(\mu +v)=r^0~~\text {a.s}. \end{aligned}$$

$$\square$$

We discuss the analysis of persistence with the help of the theorem as stated below.

### Theorem 5.2

*If* $R_0^S>1$ *and* $\alpha (\mu +v)>\varrho ^2\Lambda$ *then for any initial conditions in* $\Omega ^{*}$ *the solution of system* (1) *satisfies*

$$\begin{aligned} i_2\le \lim \inf \langle i(t)\rangle \le \lim \sup \langle i(t)\rangle \le i_1~~\text {as} t\rightarrow \infty , \text {a.s.}, \end{aligned}$$

*where*

35 $$\begin{aligned} \begin{aligned}{}&i_1=\frac{(v+\mu )\left( \gamma _1+\mu +\mu _1+\frac{\varrho ^2\Lambda ^2}{2(\mu +v)^2}\right) (R_0^S-1)}{(\gamma _1+\mu +\mu _1)\big (\alpha (v+\mu )-\Lambda \varrho ^2\big )}, \end{aligned} \end{aligned}$$

*and*

36 $$\begin{aligned} \begin{aligned}{}&i_2=\frac{(\mu +v)\left( \mu +\gamma _1+\mu _1+\frac{\varrho ^2\Lambda ^2}{2(\mu +v)^2}\right) (R_0^S-1)}{\alpha (\gamma _1+\mu +\mu _1)}. \end{aligned} \end{aligned}$$

### Proof

We can write from Eq. (28) that

37 $$\begin{aligned} \begin{aligned} \frac{\log i(t)}{t}&\le \left( \mu +\mu _1+\gamma _1+\frac{1}{2}\frac{\varrho ^2 \Lambda }{(\mu +v)}\right) \big (R_0-1\big ) \\&\quad -\left( \frac{\mu +\mu _1+\gamma _1}{\mu +v}\right) \left( \alpha -\frac{\varrho ^2 \Lambda }{(\mu +v)}\right) \langle I(t)\rangle +\frac{M(t)}{t}+\varphi (t)+\frac{\text {log}I(0)}{t}. \end{aligned} \end{aligned}$$

Using some algebraic manipulations, the above inequality may be written as

38 $$\begin{aligned} \begin{aligned} \langle i(t)\rangle&\le \frac{(\mu +v)\left( \mu +\mu _1+\gamma _1+\frac{1}{2}\frac{\varrho ^2 \Lambda }{(\mu +v)}\right) }{(\mu +\mu _1+\gamma _1)(\alpha (\mu +v)-\varrho ^2\Lambda )}\big (R_0-1\big ) \\&\quad +\frac{\mu +v}{(\mu +\mu _1+\gamma _1)(\alpha (\mu +v)-\varrho ^2\Lambda )}\left[ \frac{M(t)}{t} +\varphi (t)+\frac{\text {log}I(0)}{t}-\frac{\text {log}I(t)}{t}\right] . \end{aligned} \end{aligned}$$

Taking $\lim$ superior of both side, we obtain

39 $$\begin{aligned} \begin{aligned} \lim _{t\rightarrow \infty }\sup \langle i(t)\rangle&\le \frac{(\mu +v)\left( \mu +\mu _1+\gamma _1+\frac{1}{2}\frac{\varrho ^2 \Lambda }{(\mu +v)}\right) }{(\mu +\mu _1+\gamma _1)(\alpha (\mu +v)-\varrho ^2\Lambda )}\big (R_0-1\big )=i_1. \end{aligned} \end{aligned}$$

Again the use of Eq. (25) in Eq. (27) gives

$$\begin{aligned} \begin{aligned} \frac{\log i(t)-\log i(0)}{t}&=\frac{\alpha \Lambda }{\mu +v} -\frac{\alpha (\mu +\mu _1+\gamma _1)}{\mu +v}\langle i(t)\rangle +\alpha \phi (t)-\frac{1}{2}\varrho ^2\langle s^2(t)\rangle \\&\quad -(\mu +\mu _1+\gamma _1)+\frac{M(t)}{t}, \end{aligned} \end{aligned}$$

implies

40 $$\begin{aligned} \begin{aligned} \frac{\log i(t)-\log i(0)}{t}&\ge \frac{\alpha \Lambda }{\mu +v} -\frac{\alpha (\mu +\mu _1+\gamma _1)}{\mu +v}\langle i(t)\rangle +\alpha \phi (t) -\frac{1}{2}\frac{\varrho ^2\Lambda ^2}{(\mu +v)^2}\\&\quad -(\mu +\mu _1+\gamma _1)+\frac{M(t)}{t}. \end{aligned} \end{aligned}$$

The re-arrangement and taking $\lim$ inferior, we obtain

41 $$\begin{aligned} \begin{aligned}{}&\lim _{t\rightarrow \infty }\inf \langle i(t)\rangle \ge \frac{(\mu +v)\left( \mu +\mu _1+\gamma _1 +\frac{1}{2}\frac{\varrho ^2}{(\mu +v)^2}\right) (R_0^S-1)}{\alpha (\mu +\mu _1+\gamma _1)}=i_2. \end{aligned} \end{aligned}$$

Thus Eqs. (39) and (41) leads to the conclusion which is required $\square$

## Simulation analysis

In this section, the numerical simulation is given to verify the analytical work. First, we present a short overview of the discretization of stochastic differential equations models. We assume that

42 $$\begin{aligned} dX(t)=\alpha (t,X(t))dt+b(t,X(t))dB(t),~~~X(0)=X_0. \end{aligned}$$

Producing a sample *X*(*t*) around *t* with the utilization of the solution of the above equation, we will find *X*(*t*) over a continuous period. Making use of the notation ${\tilde{X}}_k$, $B_k$ and ${\tilde{X}}(k\Delta t)$ for simplicity instead of $B(k\Delta t)$. Thus the discretization of Eq. (42) gives

43 $$\begin{aligned} {\tilde{X}}_{\Delta t},{\tilde{X}}_{\Delta t},\ldots ,{\tilde{X}}_{N\Delta t}, \end{aligned}$$

where *N* symbolizes the time steps and $\Delta t=T/N$. It could be noted that the application of Itô-Taylor expansion leads to the stochastic Euler Maruyama (SEM) method to simulate the proposed stochastic model. We retrieve the discretized trajectory of *X*(*t*) from the Eq. (42), we may use the algorithm of Euler Maruyama:

- Simulate $\Delta B_k$ as a normal distributed random variable $N(0, \Delta t)$.
- Putting ${\tilde{X}}_0:=X_0$ and applying ${\tilde{X}}_{k+1}$ by following the formula given below
  44 $$\begin{aligned} {\tilde{X}}_{k+1}=b(k\Delta t,{\tilde{X}})\Delta B_k+\alpha (k\Delta t,{\tilde{X}}_k)\Delta t+{\tilde{X}}_k, \end{aligned}$$
  for $$\Delta B_k=B_{k+1}-B_k$$ and $$k=0,\ldots ,N-1$$.

We utilize the above technique for simulation of the corona model as stated by Eq. (1) to investigate the verification of our theoretical findings, which lead to the following system as defined by

45 $$\begin{aligned} \left\{ \begin{array}{l} s_{j+1}-s_j=\big \{\Lambda -\alpha i_js_j-(\mu +v)s_j\big \}\Delta t-\varrho i_js_j\Delta B_k,\\ i_{j+1}-i_j=\big \{\alpha i_js_j-\big (\gamma _1+\mu +\mu _1\big )i_k\big \}\Delta t+\varrho i_js_j\Delta B_k,\\ r_{j+1}-r_j=\big \{v s_j+\gamma _1 i_j-\mu r_j\big \}\Delta t, \end{array} \right. \end{aligned}$$

where $\Delta B_k$ is the random variable and are independent $N(0, \Delta t)$ normally distributed random variables. We can also write the system (45) as

46 $$\begin{aligned} \left\{ \begin{array}{l} s_{j+1}=s_j+\big \{\Lambda -\alpha i_js_j-(\mu +v)s_j\big \}\Delta t-\varrho i_js_j\Delta B_k,\\ i_{j+1}=i_j+\big \{\alpha i_js_j-\big (\gamma _1+\mu +\mu _1\big )i_k\big \}\Delta t+\varrho i_js_j\Delta B_k,\\ r_{j+1}=r_j+\big \{v s_j+\gamma _1 i_j-\mu r_j\big \}\Delta t. \end{array} \right. \end{aligned}$$

**Figure 5:**
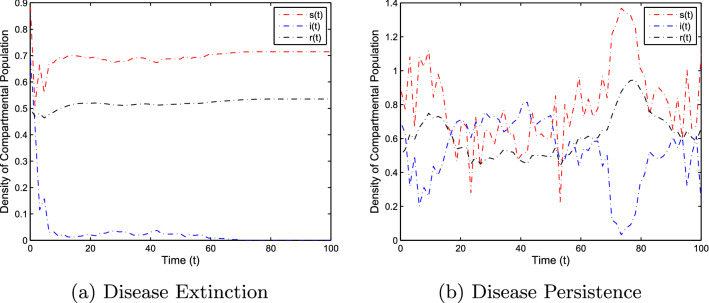
The graphs demonstrate the extinction and persistence analysis of the SARS-CoV-2 virus transmission using the proposed model (1).

**Figure 6:**
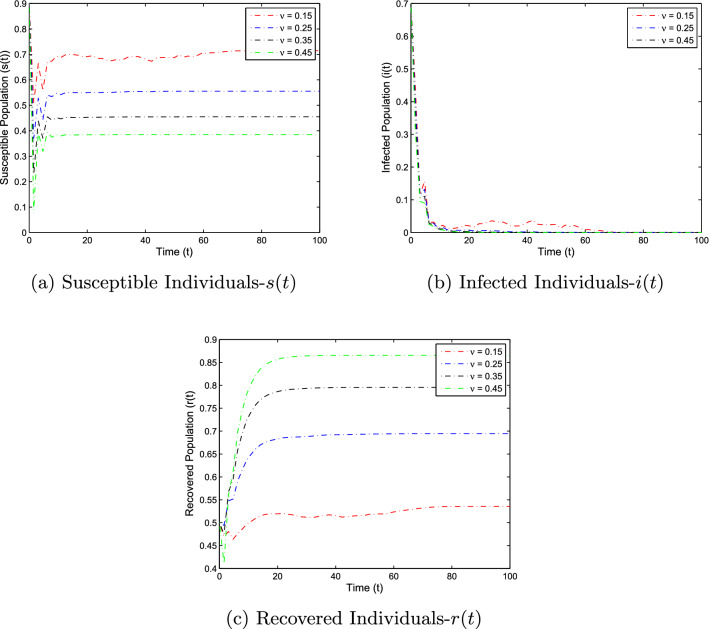
The graphs illustrate the effect of vaccination on the dynamics of susceptible, infected and recovered population in case of disease extinction. We noted that whenever the progress of vaccination are increases, the susceptible and infected population are decreases while the recovered population are increases.

**Figure 7:**
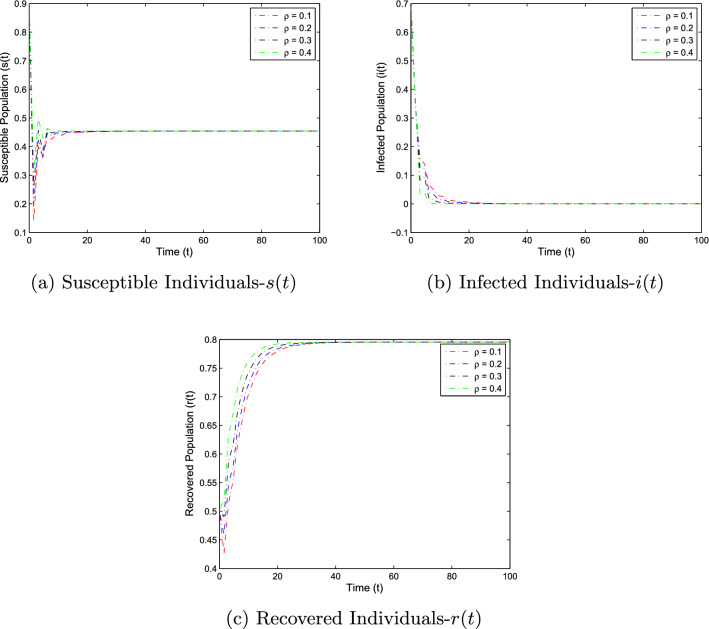
The graphs represent the temporal dynamics of the model against various values of white noise intensity in case of disease extinction, which reflects that there is no considerable effect of white noise intensity on the disease dynamics.

**Figure 8:**
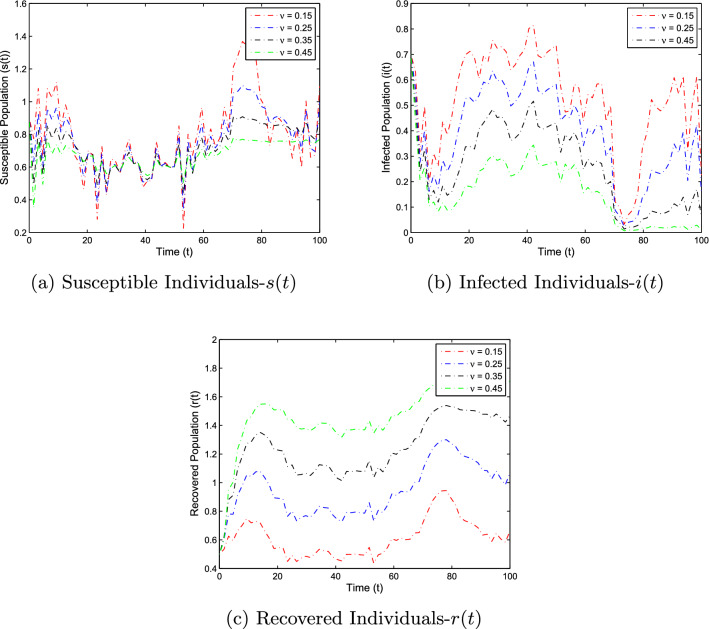
The graphs represent the effect of vaccination on the dynamics of various compartments in case of the persistence analysis of the model (1).

**Figure 9:**
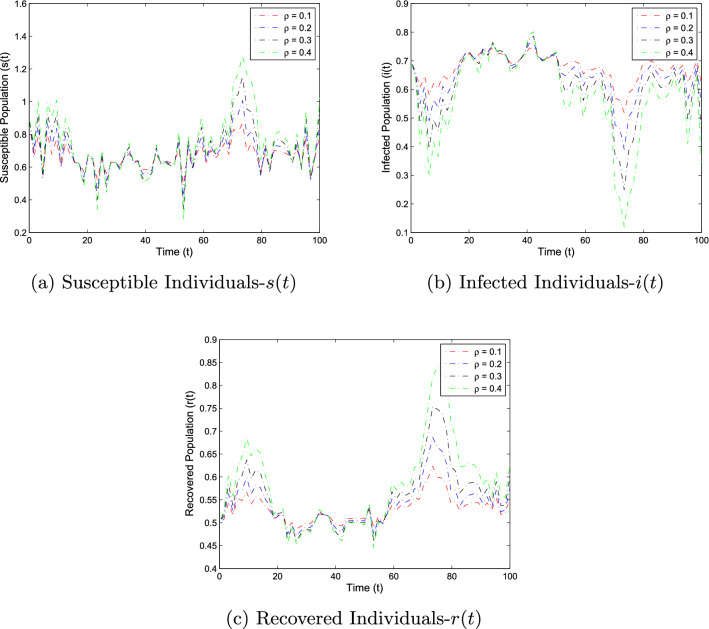
The graphs represent the effect of white noise intensity on the dynamics of the compartmental population of the epidemic problem (1) in case of disease persistence analysis.

This algorithm will be coded via Matlab, assuming feasible biological values for the parameters, as well as the initial conditions to perform the numerical simulation of the models and verify the analytical findings. We also present the influence of the stochastic process and vaccination on the novel coronavirus disease transmission. For this, purposes the two various sets of parameter values are used i.e., for extinction and persistence analysis. Let $S_1=\{\Lambda ,\alpha ,\mu ,v,\varrho , \mu _1,\gamma _1\}$ is the set of parameters whose values are assumed to be $\Lambda =0.25$, $\alpha =0.5$, $\mu =0.2$, $v=0.4$, $\varrho =0.35$, $\mu _1=0.18$ and $\gamma _1=0.01$, to perform the extinction analysis. On the other hand, the value of the parameters are taken to be $\Lambda =0.5$, $\alpha =0.6$, $\mu =0.2$, $v=0.018$, $\varrho =0.25$, $\mu _1=0.3$ and $\gamma _1=0.3$ in case of persistence analysis. Further, the initial sizes for various compartmental classes of the model are as: $(s(0),i(0),r(0))=(0.9,0.8,0.6)$. We then execute the model with the aid of the above algorithm and data and obtain the results as given in Figs. 5, 6, 7 and 8. This verifies the analytical work carried out in Theorem 5.1 and Theorem 5.2, and the effect of vaccination and white noise intensity on the dynamics of the novel disease of coronavirus. It is clear that in case of extinction analysis i.e., if $R_0^S<1$, then there will always be susceptible and recovered individuals while the infected individual vanishes as shown in Fig. 5, but if $R_0^S>1$, then there will be infected individuals and hence the disease persist as shown in the Fig. 6. Moreover, the effect of vaccination and white noise intensity on the dynamics of the compartmental population are shown in Figs. 6, 7, 8 and 9. Clearly, in both cases, vaccination and the white noise intensity parameter play a significant role. We observed that there is a powerful influence of vaccination and intensity of white noise on novel coronavirus disease transmission. It could be noted that increasing the value of these two parameters would increase the disease extinction as shown in Figs. 6 and 7, and so if the value of $\nu$ and $\varrho$ increases, the number of susceptible as well as infected individuals decreases while the number of recovered population increases. Similarly, in the case of disease persistence, $\nu$ is inversely proportional to the number of susceptible and infected individuals while directly proportional to the number of recovered individuals as depicted in Fig. 8. On the other hand, the parameter $\varrho$ is inversely proportional to the number of infected individuals while directly proportional to the number of susceptible and infected individuals, as shown in Fig. 9.

## Conclusion

The novel coronavirus disease transmissions are investigated with the help of a stochastic epidemic model under the effect of vaccination and fluctuated incidence rate. The stochastic model is formulated according to the characteristics of the disease and studies the basic fundamental axioms of well-posedness for biological and mathematical feasibility. We also discussed the detailed analysis of the extinction and the persistence analysis to identify sufficient conditions in terms of the model parameters. We simulated the model to support the analytical work and to present the impact of vaccination and the intensity of white noise. It could be noted that the stochastic process, as well as the impact of vaccination, have a positive influence on disease transmission and its elimination. We observe that the effect of vaccination and white noise intensity have a direct relation with the extinction of the disease and are inversely related to the disease persistence. It is observed that as the values of these parameters increase, then the disease extinction will increase.

In the future, we will take the extended version of the proposed model to formulate a control problem keeping in view the current scenario to find the optimal methods for the elimination of the current pandemic of the novel disease of the coronavirus.

## Data Availability

All data generated or analyzed during this study are included in this published article.

## List of symbols

*x*(*t*): Total population at time *t*
*s*(*t*): Susceptible population at time *t*
*i*(*t*): Infected population at time *t*
*r*(*t*): Recovered population at time *t*
*B*(*t*): Brownian motion
$e_0$: Disease free state
$e^{*}$: Disease endemic state
$R_0$: The basic reproductive number
$S_{\psi }$: Sensitivity index of a parameter $\psi$
$R_{+}^3$: Non-negative orthant of three dimension
$R_0^{S}$: Stochastic reproductive number
$$\tau _e$$: Explosion time
$$\tau _k$$: Stopping time
$$\phi$$: Null set
*G*: $$C^2$$-function
